# Analysis and comparison of very large metagenomes with fast clustering and functional annotation

**DOI:** 10.1186/1471-2105-10-359

**Published:** 2009-10-28

**Authors:** Weizhong Li

**Affiliations:** 1 California Institute for Telecommunications and Information Technology, University of California, San Diego, La Jolla California 92093 USA

## Abstract

**Background:**

The remarkable advance of metagenomics presents significant new challenges in data analysis. Metagenomic datasets (metagenomes) are large collections of sequencing reads from anonymous species within particular environments. Computational analyses for very large metagenomes are extremely time-consuming, and there are often many novel sequences in these metagenomes that are not fully utilized. The number of available metagenomes is rapidly increasing, so fast and efficient metagenome comparison methods are in great demand.

**Results:**

The new metagenomic data analysis method Rapid Analysis of Multiple Metagenomes with a Clustering and Annotation Pipeline (**RAMMCAP**) was developed using an ultra-fast sequence clustering algorithm, fast protein family annotation tools, and a novel statistical metagenome comparison method that employs a unique graphic interface. RAMMCAP processes extremely large datasets with only moderate computational effort. It identifies raw read clusters and protein clusters that may include novel gene families, and compares metagenomes using clusters or functional annotations calculated by RAMMCAP. In this study, RAMMCAP was applied to the two largest available metagenomic collections, the "Global Ocean Sampling" and the "Metagenomic Profiling of Nine Biomes".

**Conclusion:**

RAMMCAP is a very fast method that can cluster and annotate one million metagenomic reads in only hundreds of CPU hours. It is available from .

## Background

The emerging field of metagenomics enables a more comprehensive understanding of environmental microbial communities [1-9]. However, metagenomic data consists of enormous numbers of fragmented sequences that challenge data analysis methodologically and computationally. To address these challenges, new methods and resources have been developed, such as simulated datasets[10], IMG/M[11], CAMERA[12], MG-RAST[13], taxonomy tools[14,15], statistical comparison[16], functional diversity analysis[17], binning [18-20] and so on.

The Rapid Analysis of Multiple Metagenomes with a Clustering and Annotation Pipeline (RAMMCAP) presented herein aims to address the particular computational challenges imposed by the huge size and great diversity of metagenomic data. The primary goal is to significantly reduce the computational effort in sequence comparison, as large-scale comparison of metagenomic sequences has become extremely time-consuming. For example, the protein analysis of the Global Ocean Sampling (GOS) study[2] took more than one million CPU hours.

Metagenomic datasets may contain many novel genes that don't show any homology to existing genes. For example, only ~10% of the sequences in the "Metagenomic Profiling of Nine Biomes" (BIOME) study [9] match known functional genes. Novel genes in metagenomic datasets have not been used in many studies with homology-based gene prediction and analysis, so the second goal of RAMMCAP is to explore whole datasets and make use of the novel sequences. Because the *ab initio *gene finding approaches developed for complete genomes work poorly with fragmented DNA sequences, recently, several new gene prediction methods were developed for short DNA sequences with high sensitivity and specificity, such as Metagene[21], MetageneAnnotator[22], and Neural Networks[23]. In RAMMCAP, ORFs are called with either Metagene or simple six reading frame translation; both methods can identify novel genes.

Since more and more metagenomes will be available in the future, the third goal of RAMMCAP is to provide a new way to compare metagenomes from various environmental conditions and to identity and visualize the statistically significant differences between metagenomes.

In this paper, RAMMCAP was implemented and applied to the two largest metagenomic collections. The first set, GOS [1,2], features 7.7 million ~800 base Sanger reads from 44 samples. A second, the Biomes [9] set, has 14.6 million ~100 base 454 reads from 45 microbiomes and 42 viromes samples. With moderate computational effort, RAMMCAP can quickly analyzed these huge datasets and obtained many novel results that could not be achieved by other existing methods.

## Results and Discussion

### Implementation

**Figure 1:**
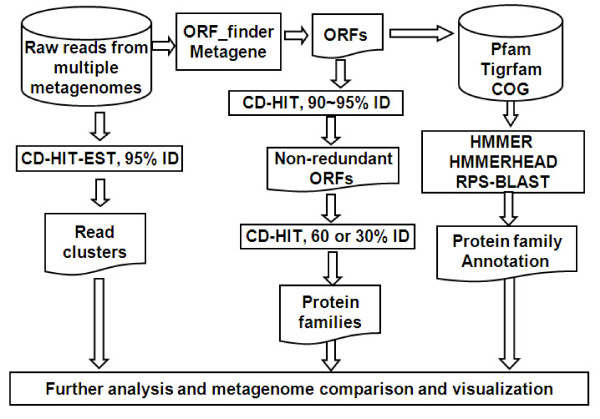
**Metagenomic data analysis pipeline RAMMCAP**.

RAMMCAP is illustrated in Figure 1. Cluster analysis is a key approach in this pipeline. Our previous ultra-fast sequence clustering algorithm CD-HIT [24-26] was modified to handle large metagenomic datasets. Using the DNA version of CD-HIT, the metagenomic reads from one or more metagenomes are clustered together at 95% sequence identity over 80% of length (clustering parameters can be adjusted by users) to identify clusters of unique genomic sequences, referred to as read clusters. It takes ~1 hour to cluster a million 200 base reads.

ORFs are collected from sequence reads with ORF_finder, a ORF calling program implemented here by six reading frame translation in a similar way as the GOS study[2]. Within each reading frame, an ORF starts at the beginning of a read or the first ATG after a previous stop codon; it ends at the first stop codon or the end of that read. The minimal length of ORFs can be specified by users. ORFs can also be called from sequence reads with program Metagene[21]. Since these sequence reads are short, a predicted ORF maybe a portion of a complete ORF. An ORF may also be a translation from a non-coding frame: such an ORF is called a spurious ORF, as defined in the original GOS study [2]. The GOS study also introduced a spurious ORF detection method using nonsynonymous to synonymous substitution test, which is available along with a recent GOS clustering study [27]. This method is not integrated within RAMMCAP, but it can be used independently to identify the spurious ORFs predicted here.

ORFs are first clustered at 90-95% identity to identify the non-redundant sequences, which are further clustered to families (ORF clusters) at a conservative threshold, so that each cluster contains sequences of the same or similar function. A 30% sequence identity indicates significant similarities for full-length proteins. Since ORFs from Sanger reads are long enough, so they are clustered at 30% identity over 80% of ORF length. ORFs from 454 reads are much shorter; they are clustered at 60% identity over 80% of ORF length. ORF clusters are used for functional studies. The size of an ORF cluster is the number of its non-redundant sequences. For one million ORFs, it takes a few CPU hours to cluster at 60% identity and ~100 CPU hours at 30% identity.

The clustering method in RAMMCAP is quite different from the clustering method in the GOS study [2] and its incremental update [27], which generated core clusters by all-against-all BLAST search and then merged core clusters into final clusters using sequence profile methods. The final clusters in the GOS study are large and contain sequences of very remote similarities, whereas the clustering method employed here only tries to generate very conservative clusters.

ORFs are annotated from Pfam and Tigrfam with HMMER[28] (accelerated with Hammerhead[29]), and from COG with RPS-BLAST[30]. Hits must be with e-values ≤ 0.001, and meet the significant scores in case of HMMER searches. This annotation process only takes ~100 CPU hours for one million ORFs.

Optionally, ORF annotation may be performed quickly by running only the representative sequence of each ORF cluster and then transferring the annotation to other members in that cluster. But transferred annotation may be wrong in some cases, for example, where the target ORF has fewer domains than the source ORF (the representative). Since the annotation process is very fast, it is preferable to run all the ORFs for more accurate annotation.

### Statistical comparison of metagenome

In many metagenomic projects, multiple samples from different environmental conditions were studied. This manuscript describes a novel way to compare metagenomes and visualize their differences. Since sequences from multiple metagenomes are clustered into families or classified into reference families (Pfam, Tigrfam, or COG), metagenomes can be compared by their occurrence profiles across all the families or selected families of interests.

Here, an occurrence profile coefficient, r_AB _= N_A ∩ B_/N_A ∪ B_, is defined as the similarity measure between two metagenomes A and B. N_A ∩ B _is the number of families that are found in both A and B above a minimal occurrence cutoff (defined later) without significant difference. N_A ∪ B _is N_A ∩ B _plus the number of families that occur in one metagenome significantly higher than in another metagenome. The value of r_AB _is between 0-1, with 0 representing no overlap and 1 indicating a perfect match between A and B.

Let *N*_*A *_and *N*_*B *_be the number of sequences in A and B, let *H*_*A *_and *H*_*B *_be the number of sequences that occur in family H. One question is whether the difference between *H*_*A *_and *H*_*B *_is statistically significant. Rodriguez-Brito *et al *introduced a method to test such statistical significance of differences between two metagenomes [16]. Rodriguez-Brito's method used a large amount (in order of 10^5^) of simulations by randomly picking a certain number of sequences from A, B, and A+B to generate distributions and analyze it, so it is very time-consuming.

**Figure 2:**
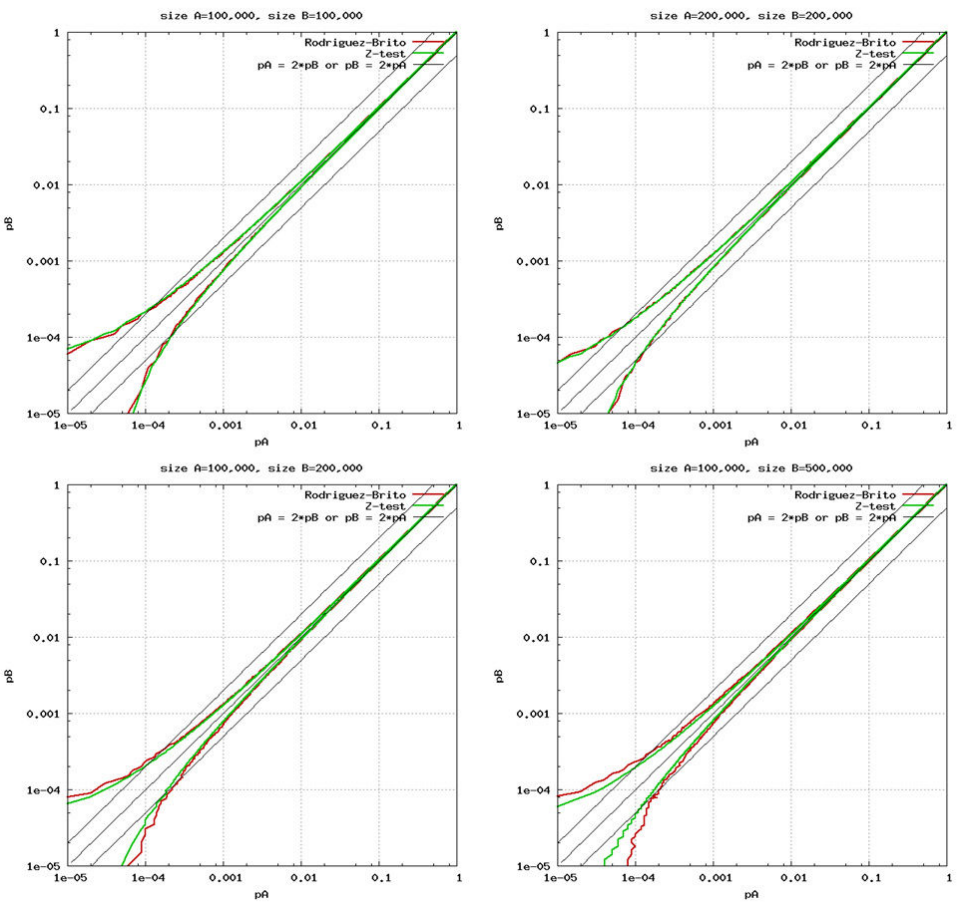
**Comparison between Rodriguez-Brito's method and z test method**. The *x*-axis and *y*-axis are occurrence rate *P*_*A *_and *P*_*B *_of two samples A and B. The 4 plots are made with different combination of sample size *N*_*A *_and *N*_*B *_as indicated in each plot. Red lines and green lines are calculated with Rodriguez-Brito's method and *z *test method respectively. Difference of A and B outside the area enclosed by a pair of red (or green) lines is statistically significant at 0.95 confidence level. This figure shows that when *P*_*A *_and *P*_*B *_become big enough (such as >0.001), a very small difference between them will be counted as significant.

In this paper, the ***z ***test for two independent proportions[31] is adopted to test the statistical significance of differences between two metagenomes. Given *N*_*A*_, *N*_*B*_, *H*_*A*_, and *H*_*B*_, there are three occurrence rates *P*_*A *_= *H*_*A*_/*N*_*A*_, *P*_*B *_= *H*_*B*_/*N*_*B*_, and *P *= *(H*_*A*_*+H*_*B*_*)/(N*_*A*_*+N*_*B*_*)*. The statistical significance between A and B can be described by:



This method just needs calculation of a single equation, but it produces near identical results as the Rodriguez-Brito's method. Comparisons between the Rodriguez-Brito's method and the ***z ***test method under several different combinations of *N*_*A*_, *N*_*B*_, *H*_*A*_, and *H*_*B *_is shown in Figure 2.

In this manuscript, *H*_*A *_is considered significantly higher than *H*_*B *_if both (1) the ***z ***score satisfied a user defined confidence level such as 0.95, and (2) *P*_*A *_≥ *f *× *P*_*B*_, where f (*f*>1) is also a user defined parameter, called significant factor.

The statistical significance cannot be established at very low occurrences, so the low occurrence families are excluded from N_A ∩ B _in calculation of r_AB_. The minimal occurrence cutoff of a family H within metagenome A is defined as the minimal number of *H*_*A *_to produce a significant ***z ***score when A is compared to another metagenome B where *N*_*A *_= *N*_*B *_and *H*_*B *_= 0. It can be obtained that:



Since 2*N*_*A *_>>***z***^2^, the minimal cutoff of *H*_*A *_is 4 at 0.95 confidence level (*z *= 1.96), and 7 at 0.99 confidence level (*z *= 2.58).

The occurrence profile coefficients are calculated for all the metagenome pairs, and the output matrix is used to hierarchically cluster the metagenomes.

### Performance of ORF prediction

ORF_finder and Metagene [21] were evaluated with simulated metagenomic reads generated with MetaSim software[32] from the completed microbial genomes released between January and May 2009 from NCBI. Four datasets (Sim100, Sim200, Sim400 and Sim800) of 1 million reads each with average length of 100, 200, 400 and 800 bases were generated to simulate the current sequencing techniques. The error rates for both 454 (>3%) and Sanger (1~2%) defined by MetaSim are much higher than the reported error rates [33,34], so the exact model with default parameters were used in MetaSim for all simulated datasets. If a simulated read overlaps with an annotated ORF by NCBI at least 30 amino acid, the overlapped part is used as a true ORF. A predicted ORF by either ORF_finder or Metagene is a true-positive if at least 50% of it overlaps with a true ORF within the same reading frame. The ratios of true-positives relative to all true ORFs (sensitivity) and to predicted ORFs (specificity) were used as a performance measure (Table 1). Four ORF cutoff lengths (30, 40, 50, and 60 amino acids) were applied for ORF_finder. This analyses show that Metagene has high sensitivity and specificity (~94% and ~92%) for reads of at least 200 bases. But its sensitivity drops to 59% for 100 base sequences. ORF_finder has very high sensitivity (>99%) and very low specificity (<37%) at 30 amino acid cut off length; a longer cutoff length produces a higher specificity but lowers the sensitivity.

**Table 1:** Sensitivity and specificity of Metagene and ORF_finder on simulated metagenomic datasets

	**Sensitivity/specificity (%)**			
	
**Datasets** **Method**	**Sim100**	**Sim200**	**Sim400**	**Sim800**
Metagene	59.07/92.58	94.31/92.32	93.84/91.91	94.02/93.25
ORF_finder, cut off = 30aa	99.93/36.56	99.74/36.73	99.78/34.49	99.92/32.83
ORF_finder, cut off = 40aa		96.74/46.65	97.89/43.81	99.13/41.54
ORF_finder, cut off = 50aa		91.65/54.82	94.04/52.51	96.88/50.21
ORF_finder, cut off = 60aa		73.66/61.27	88.94/59.84	93.34/57.98

### Testing on clustering

**Figure 3:**
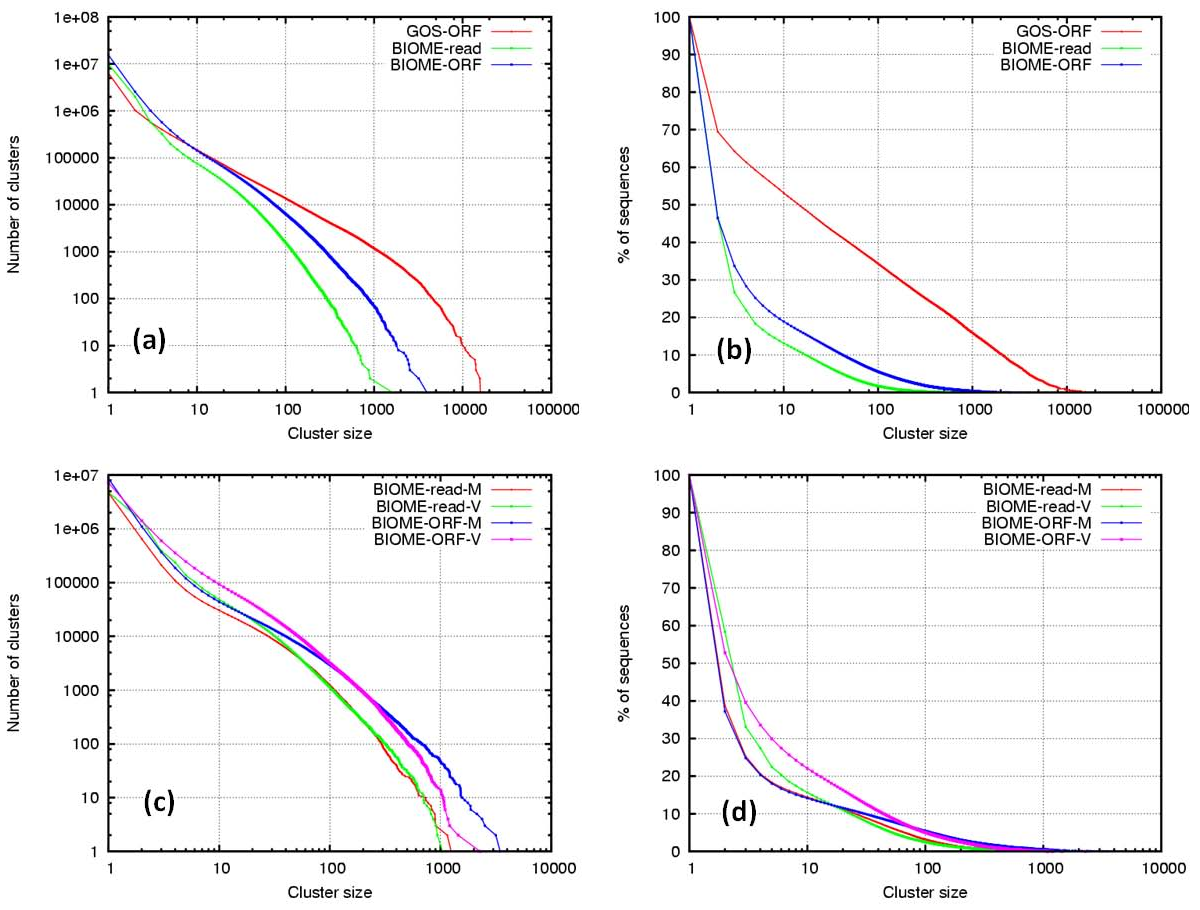
**Distribution of clusters and sequences by cluster size**. The *x*-axis is the cluster size *X*. The *y*-axis in left figures (a and c) is the number of clusters of size at least *X*; the *y*-axis in right figures (b and d) is the percentage of total sequences included in the clusters of size at least *X*. Clustering analyses were also made separately for the microbiomes and the viromes. So, together there are seven clustering experiments: GOS-ORF, BIOME-read, BIOME-ORF, BIOME-read-M, BIOME-read-V, BIOME-ORF-M, and BIOME-ORF-V (where M and V stand for microbiomes and viromes).

RAMMCAP was applied to GOS[2] and BIOME[9], the two largest metagenomic collections. Prior to the full development of RAMMCAP, we clustered the 17.4 million GOS ORFs released by the original GOS study [2] at 30% identity and published the detailed clustering results [35]. Here the BIOME reads and BIOME ORFs, which have 14.6 and 24.6 million sequences respectively, were clustered at 95% and 60% identity (Figure 3). The previous GOS clusters were added in Figure 3 to show the difference between GOS and Biome (Figure 3a, b).

GOS ORFs have more than 1000 clusters that contain ≥ 1000 non-redundant sequences; BIOME ORFs have less than 100 such clusters. About 70% GOS ORFs, 46% BIOME reads, and a similar percent BIOME ORFs are found in non-singleton clusters. Within the BIOME datasets (Figure 3c, d), the microbiomes have more large clusters compared to viromes; suggesting that microbial sequences are more conserved than viral sequences.

Clustering analysis is a powerful tool to recover protein families and to discover the novel ones; it helps to recognize spurious ORFs. Clustering tends to put real ORFs into large clusters and leaves spurious ones in small or singleton clusters because spurious ORFs have more random features. If the ORFs with Pfam, Tigrfam, or COG matches are considered true ORFs, then 93% of these true GOS ORFs are found in clusters of size ≥ 10, which is only 1.3% total GOS clusters; here cluster size is the number of non-redundant sequences in a cluster. Further, 28% of the true BIOME ORFs are in 1.0% of top clusters of size ≥ 5. Many large clusters without any homology to known proteins are found, which may shed light on novel families of environment specific functions.

### Validation of clustering quality

ORFs called from metagenomic reads are short and fragmented. In addition, errors such as frame shifting and wrong gene boundary may occur due to sequencing errors. Therefore a conservative threshold is used in producing ORF clusters to ensure that a cluster contains sequences of the same or similar function. The quality of clustering was evaluated with Pfam, the manually curated classification of protein families. The domain sequences in Pfam models (release 22.0) were extracted from the alignments and were clustered at 30% identity using the clustering protocol in RAMMCAP.

**Figure 4:**
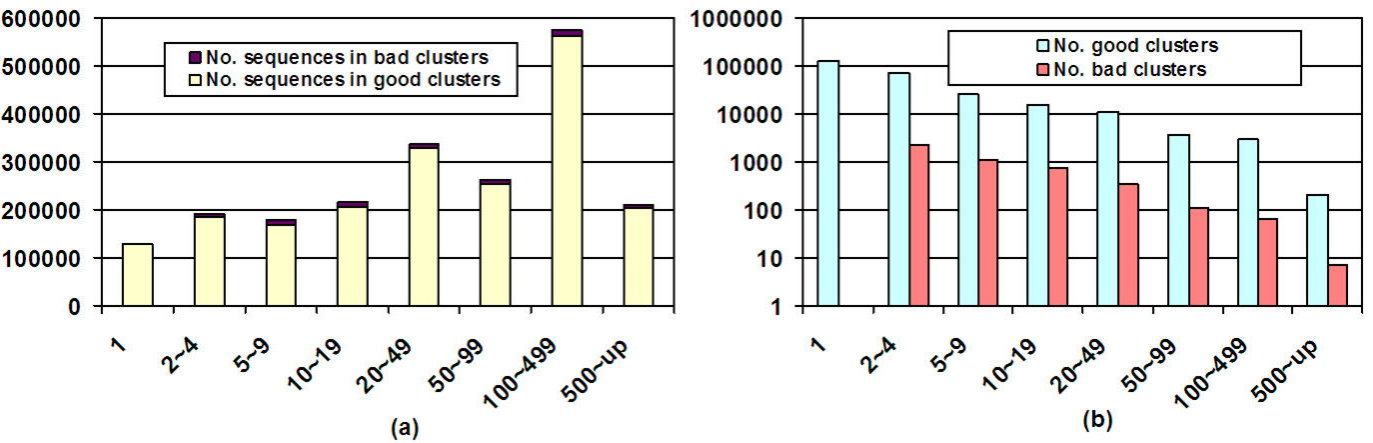
**Distribution of clusters of Pfam sequences**. The *x*-axis is cluster size. The *y*-axis in (a) is the number of sequences, and the *y*-axis in (b) is the number of clusters.

Not all the Pfam sequences were used. Very short Pfam families <30 amino acids were excluded because most of these families were built by sequence patterns rather than similarities. Some Pfam families are overlapping, for those families, short ones were excluded. Since Pfam families were built with very sensitive HMM models, the sequences within same families can be very diverse, even at sequence identities very much below 30%. Therefore it is anticipated that divergent sequences from a large Pfam family may be placed in separate clusters. The goals of clustering are: (1) to generate homogeneous clusters whose sequences are from the same Pfam families, (2) to cover most sequences in a small number of large clusters. The distributions of clusters of Pfam domain sequences are shown in Figure 4. A cluster is considered good if >95% of its members are from the same Pfam family. It is observed that most sequences (>97%) are in good clusters (Figure 4a), which are ~30 times more than bad clusters (Figure 4b). Although there are singleton clusters, but non-singleton clusters still cover 94% of sequences, and clusters of size ≥ 5 cover 85% of sequences.

### Testing on clustering-based comparison of metagenomes

**Figure 5:**
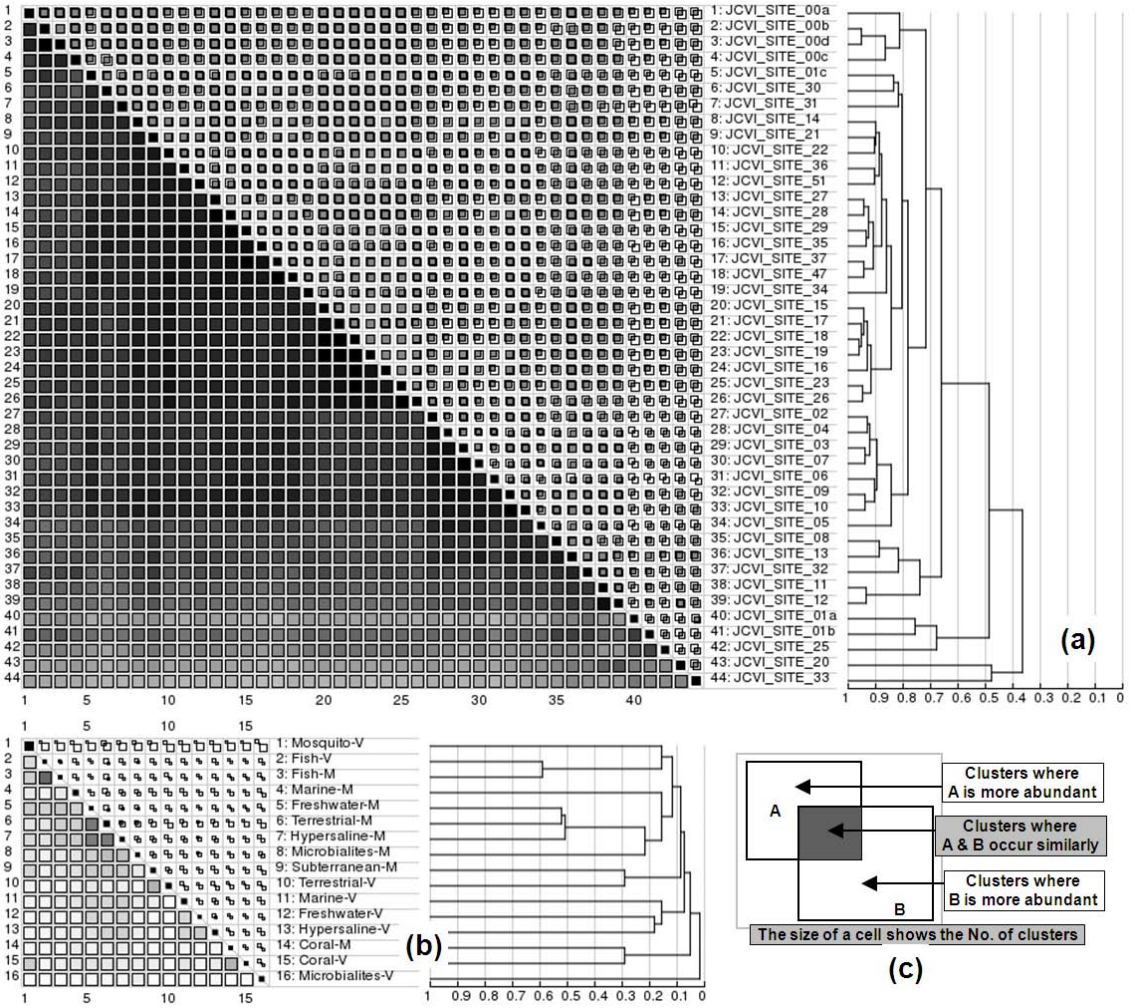
**Similarity matrices of metagenomes**. Squares along the diagonal represent the number of clusters where a sample occurs. Grayscale squares below the diagonal represent the occurrence profile coefficients r_AB _between two samples with a darker color indicating a greater similarity. Cells above the diagonal show the unique and overlapping clusters, explained in **(c)**. Hierarchical clustering of samples based on the matrix is shown with vertical gridlines indicating the value of the coefficient where two nodes are merged. Matrices are made for GOS ORF clusters **(a) **and BIOME ORF clusters **(b) **with significant a factor *f *= 2 at 0.95 confidence level. The BIOME samples are grouped by biome type, such as Coral-M, which stands for coral microbiomes sample.

Statistical comparisons of GOS and BIOME metagenomes based on the occurrence profile coefficient calculations using results of clusters are shown in Figure 5. The GOS samples show great overlaps, but all unique samples are classified as outliers, such as GS033 (hypersaline), GS020 (fresh water), and GS025 (reef, different filter size), from other marine samples. The sample-specific clusters may shed light on functional aspects related to the environment for further studies. The BIOME samples intersect much less, but notable overlaps are found between pairing samples, such as Fish-M vs Fish-V, and Coral-M vs Coral-V. The differences between GOS and BIOME samples reflect that the BIOME samples are more diverse.

### Testing on protein family-based comparison of metagenomes

**Figure 6:**
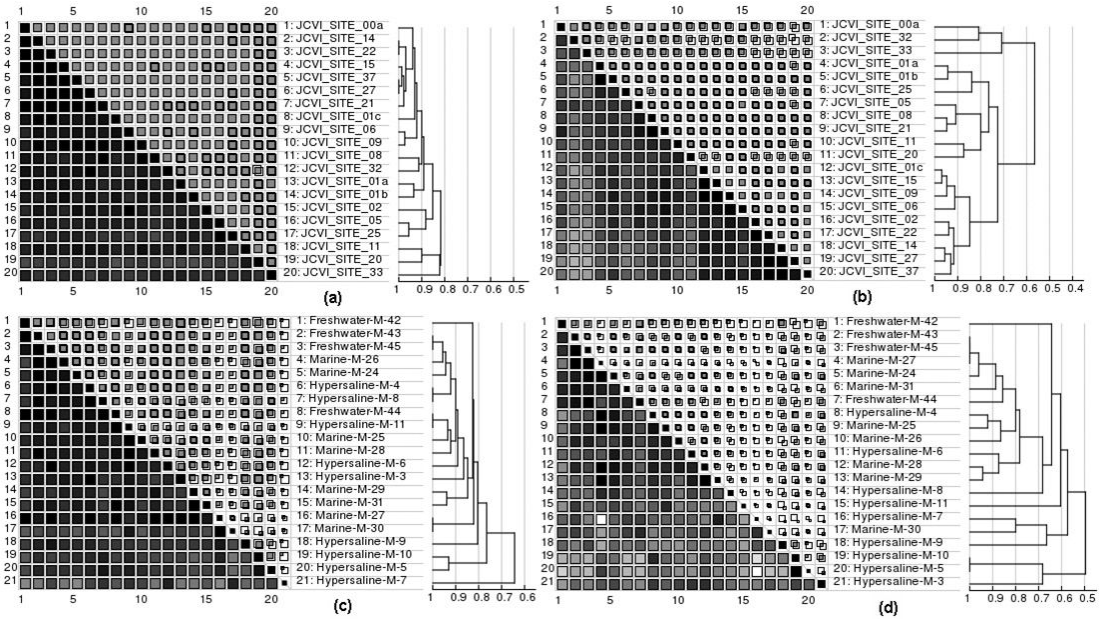
**Similarity matrices of metagenomes based on families of two COG classes**. Matrices are for GOS on COG class F **(a)**, GOS on class T **(b)**, BIOME on F **(c)**, and BIOME on T **(d) **respectively with significant a factor *f *= 2 at 0.95 confidence level. Because GOS samples are microbial marine samples, only the microbial (non-viral) water samples from the BIOME data was used. Further, a representative subset from GOS samples was selected so that the figures of GOS and BIOME are similar in size.

Annotations from Pfam, Tigrfam, and COG are used for metagenome comparison in a similar way that is applied in clusters. These analyses show over- and under-represented families between samples. The protein families of these three databases are all organized into super families: clans in Pfam, role categories in Tigrfam, and functional classes in COG. Therefore, metagenome comparison can be made under a specific super family of interest, which is a unique feature of this study. Samples were compared systematically under all super families and many significant differences were found. Here we show a few interesting examples (Figure 6). GOS samples share an extremely similar occurrence profile across the 86 families of COG class F (nucleotide transport and metabolism), which suggests this class is highly conserved across the world's ocean. The least conserved class, aside from functional unknown classes and a few tiny classes, is T (Signal transduction mechanisms). Similar observations were obtained for Biome samples, but the intrinsic diversity of BIOME sequences introduces more non-overlapping families.

## Conclusion

The CPU time for clustering GOS ORFs, BIOME reads, and BIOME ORFs were 9600, 125, and 513 hours, respectively. GOS ORFs cost relatively more, but still two orders of magnitude less than the original GOS study[2]. The annotation for GOS ORFs and BIOME ORFs took 3800 and 1560 hours. Through clustering analysis, many novel families can be identified and can be used in metagenome comparison. The RAMMCAP software and pre-calculated results are available at .
